# Selective vulnerability of stellate cells to gut dysbiosis: neuroanatomical changes in the medial entorhinal cortex

**DOI:** 10.3389/fnana.2025.1589287

**Published:** 2025-08-13

**Authors:** Ayishal B. Mydeen, Mohammed M. Nakhal, Faheema Nafees, Reem Almazrouei, Rasha Alkamali, Mahra Alsulaimi, Omar Aleissaee, Abdulrahman Alzaabi, Mohamed Alfahim, Hamad Almansoori, Shamsa BaniYas, Shaikha Al Houqani, Marim Elkashlan, Safa Shehab, Mohammad I. K. Hamad

**Affiliations:** Department of Anatomy, College of Medicine and Health Sciences, United Arab Emirates University, Al Ain, United Arab Emirates

**Keywords:** medial entorhinal cortex, microbiota-gut-brain axis, dysbiosis, stellate cells, pyramidal island cells, dendritic morphology

## Abstract

**Introduction:**

The gut microbiota plays a critical role in regulating brain structure and function via the microbiota–gut–brain axis. Antibiotic-induced gut dysbiosis (AIGD) has been linked to neuroanatomical changes and cognitive deficits. However, its impact on neuronal morphology in layer II of the medial entorhinal cortex (mECII), a region central to spatial memory, remains poorly understood. This study examines how AIGD affects dendritic architecture in mECII stellate and pyramidal island cells.

**Methods:**

Mice received a broad-spectrum oral antibiotic cocktail to induce AIGD. Gut microbiota composition was analyzed using 16S rRNA sequencing. Golgi-stained neurons in mECII were assessed for dendritic complexity via Sholl analysis. Iba1 staining evaluated microglial activation in mECII. Intestinal sections were stained with NeuN and CD8 to assess enteric neuron density and inflammation. Microbial abundance was correlated with dendritic parameters.

**Results:**

AIGD resulted in significant dysbiosis, including depletion of butyrate-producing taxa (*Roseburia*, *Faecalibacterium*) and enrichment of proinflammatory bacteria (*Clostridium*, *Salmonella*, *Enterococcus*). Stellate cells showed marked dendritic atrophy, while pyramidal island cells were unaffected. Dendritic complexity positively correlated with *Roseburia hominis* and negatively with *Enterococcus faecalis*. No microglial activation was detected in mECII, but CD8 + T-cell infiltration increased in the gut without changes in NeuN-labeled enteric neurons.

**Discussion:**

These findings suggest AIGD selectively alters mECII stellate cell morphology through peripheral immune signaling or microbial metabolites, independent of local microglial activation. This study highlights the role of gut microbiota in shaping neuronal architecture and supports microbiome-targeted strategies to counteract dysbiosis-associated neuroanatomical changes.

## 1 Introduction

The medial entorhinal cortex layer II (mECII) plays a pivotal role in spatial navigation and memory through its robust connections with the hippocampus and other cortical and subcortical regions (Kerr et al., 2007; Agster and Burwell, 2013; Witter et al., 2017, p. 201; Tukker et al., 2022). mECII neurons, including stellate cells (stellate cells) and pyramidal island cells, exhibit distinct morphologies, physiological properties, and projection targets, making them essential for contextual and spatial memory (Klink and Alonso, 1993; Fuchs et al., 2016; Kitamura, 2017; Hamad et al., 2024). Stellate cells, which project to the dentate granule cells and CA3 pyramidal cells, are particularly involved in forming novel context representations, while island cells project to CA1 and interneurons but are not context-specific in their activity (Kitamura et al., 2015; Ray and Brecht, 2016). Recent studies emphasize the importance of the mECII cells in memory and spatial navigation, with disturbances in these cells linked to neurodegenerative conditions, including Alzheimer’s disease (Kobro-Flatmoen et al., 2016; Chen et al., 2023). Despite this, the underlying mechanisms influencing the development and maintenance of dendritic morphology in these cells remain poorly understood. Dendrites play a key role in the integration and transmission of information within the nervous system. The formation and maturation of dendrites are crucial for establishing neural connections (Valnegri et al., 2015). Dendrite development is a critical process that is susceptible to defects, which can impede the formation of neuronal circuits and the subsequent information processing between neurons. The mechanisms that regulate dendritic growth are complex and involve the interplay of intrinsic and extrinsic signaling molecules (Hamad et al., 2023).

The gut microbiome, through the microbiota-gut-brain axis (MGBA), significantly impacts brain function, influencing cognitive processes, emotional regulation, and memory (Nakhal et al., 2024; Yassin et al., 2025). This complex system communicates bidirectionally between the central nervous system (CNS) and the enteric nervous system (ENS), with the vagus nerve serving as the primary pathway (Kaelberer et al., 2018). Disruptions in the gut microbiota, such as AIGD, can lead to systemic inflammation and compromise CNS function, contributing to disorders such as anxiety, depression, and memory deficits (Wang et al., 2021). Germ-free mice, which completely lack microbiota, exhibit profound differences in immune system development and function compared to conventionally colonized animals. These mice display impaired dendritic cell migration and altered immune responses, underscoring the critical role of gut microbes in shaping neuroimmune interactions (Round and Mazmanian, 2009). Such immunological deficiencies further highlight how microbial composition can influence brain development and neuronal structure, supporting the focus on microbiota alterations in our study. Furthermore, gut-derived metabolites like short-chain fatty acids (SCFAs) are critical in modulating brain activity, with butyrate shown to impact neurotransmitter synthesis and BDNF expression (Strandwitz, 2018; O’Riordan et al., 2022). AIGD, induced by antibiotics, leads to increased intestinal permeability and altered microbial composition, which in turn influences brain function and behavior (Bonaz et al., 2018; Nagu et al., 2021). Studies have shown that AIGD impairs memory and induces neuroinflammation through changes in neurotransmitter systems and BDNF expression (Fröhlich et al., 2016; Takahashi et al., 2024). Importantly, alterations in dendritic morphology, particularly in the hippocampal and cortical neurons, have been observed in dysbiosis models, indicating that changes in the gut microbiome can directly affect neuronal architecture (Silva et al., 2020; Guo et al., 2022). Given the critical role of the mECII in spatial navigation and memory, and the potential influence of gut microbiota on brain architecture, this study seeks to determine how AIGD affects the dendritic morphology of stellate and island cells in the mECII. Understanding these interactions could provide valuable insights into the mechanisms linking gut health to cognitive function and spatial memory.

## 2 Materials and methods

### 2.1 Antibiotic treatment and sample collection

**Figure 1:**
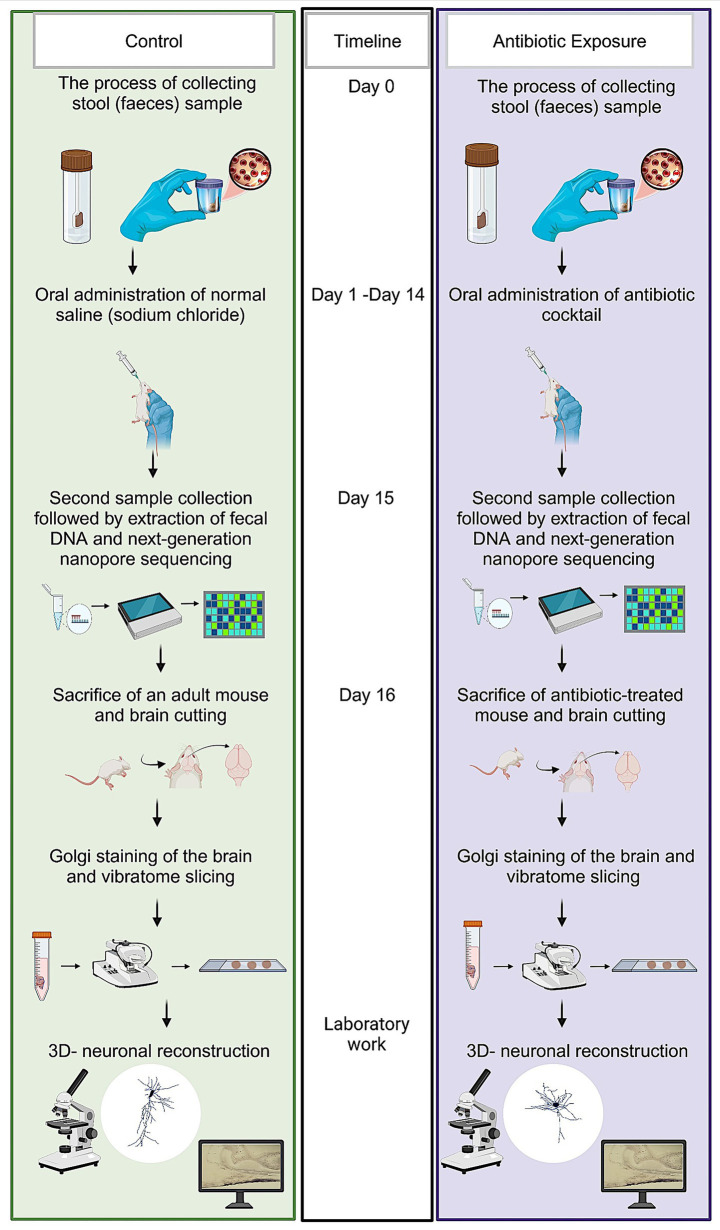
Schematic representation of experimental protocol. The sequence of steps outlined in this figure details the experimental workflow employed to investigate the effects of microbiome modulation on neuronal structure and function.

All experiments were conducted on postnatal day 90 (P90) male mice to avoid potential hormonal variations associated with the female estrous cycle. During the experimental period, animals were single-housed to enable accurate tracking of individual fecal sampling and microbiome sequencing. A summary of the entire experimental procedure is provided in (Figure 1). The experimental group received a freshly prepared mixture of blood–brain barrier impermeant antibiotics, containing 1.6 mg/ mL of vancomycin (Vancolon, Julphar, UAE), 0.83 mg/ mL of clindamycin (Vianex S. A, Greece), and 4.8 mg/mL of meropenem (Meronem, Pfizer, United States). The control group received vehicle (normal saline). Mice were dosed daily with 0.15 mL of the mixture or vehicle for 14 consecutive days by oral gavage in accordance with the Washington State University Institutional Animal Care and Use Committee protocol (Turner et al., 2011). Fecal content of both groups was obtained pretreatment (Day-0) and post treatment (Day-15). The selection of antibiotic cocktail was based on comparable research in mice and rats which have previously employed similar treatment procedures, resulting in the development of substantial alterations in the microbiome (Soares et al., 2017; Xie et al., 2019; Hertz et al., 2020; Lee et al., 2020). A total of *n* = 24 male mice were used in this study. Animals were randomly assigned to either the antibiotic-treated group (AIGD, *n* = 12) or the control group (*n* = 12). For each group, subsets of animals were allocated for microbiome analysis (*n* = 6 per group) and Golgi-Cox morphological analysis (*n* = 6 per group).

### 2.2 Extraction and quantification of DNA

The isolation of DNA from the fecal samples was conducted under aseptic conditions using the QIAamp PowerFecal Pro DNA kit (Qiagen, Hilden, Germany), in accordance with the manufacturer’s instructions. The concentration was quantified using a Qubit 4 fluorometer (Thermo Fisher Scientific, Waltham, MA, United States) in conjunction with the Qubit 1X dsDNA High Sensitivity Assay Kit (Invitrogen, Life Technologies Corporation, Eugene, OR, United States).

### 2.3 Golgi-Cox staining of neuronal dendrites

The tissue preparation and staining processes were conducted in accordance with the manufacturer’s guidelines, utilizing the FD Rapid GolgiStain kit (Du, 2019) (FD NeuroTechnologies; PK401 Cell Systems Biology). Following the decapitation of the mice, the brains were extracted and thoroughly rinsed with water. Thereafter, they were submerged in the impregnation solution (equal volumes of solutions A and B). Subsequently, after a period of 2 weeks, the brains were transferred to solution C for an additional week. A vibratome was then utilized to excise 50 μm parasagittal sections from both hemispheres, extending to the medial extent of the MEC, which was identified by the absence of the thick white band surrounding the external capsule. The preparation of a maximum of 16 sections containing the MEC from both hemispheres was conducted for each animal. The brain slices were subjected to a rehydration process using milli-Q water, followed by an immersion in solutions D and E for a period of 10 min to facilitate staining. Thereafter, the slices were rinsed once more in milli-Q water, dehydrated, and mounted using a water-based mounting medium.

### 2.4 3D neuron reconstruction of stellate cells and island cells

Golgi-stained cells were reconstructed using the Neurolucida system (Micro Brightfield). The images were taken at 1,000x magnification. A comparative analysis of stellate and interneurons reveals a significant disparity in the number of primary dendrites. Stellate cells exhibit a higher density of these structures, with a greater abundance in spines. Additionally, stellate cells’ axons demonstrate a distinct pattern of extension, deviating from the typical local branching observed in interneurons. This deviation is characterized by a preferential extension toward the white matter, suggesting a distinct functional role in the nervous system. The quantification of stellate cell morphological parameters was achieved by calculating the mean dendritic length (the total number of dendritic lengths divided by the number of primary dendrites), the mean number of dendritic segments (the total number of dendritic segments divided by the number of primary dendrites), and the number of primary dendrites. MECII island cells are characterized by the presence of one apical dendrite that originates from the cell soma, in conjunction with multiple small basal dendrites. A considerable abundance of spines is observed on the dendrites of island cells. The quantification of island cell morphological parameters was performed through the calculation of apical dendritic length and mean basal dendritic length (the total number of dendritic lengths divided by the number of primary dendrites). Furthermore, total apical dendritic segments and mean number of basal dendritic segments (the total number of basal dendritic segments divided by the number of primary dendrites) were calculated. A Sholl analysis was conducted on the number of dendrite intersections at 10 μm interval distance points starting from the soma. This analysis was performed to identify the area where dendritic complexity changed (Hamad et al., 2021, 2024).

### 2.5 Next, generation sequencing

The 16 s rRNA gene (1.5 kb) was amplified using the 16S Barcoding kit 24 V14 SQK-16S114.24 (Oxford Nanopore Technologies, Oxford, United Kingdom) and LongAmp Hot Start Taq 2X Master mix (New England Biolabs, United Kingdom) with 10 ng of genomic DNA per sample. A volume of 10 μL of each 16 S barcode was transferred into the respective sample tubes. The amplification of DNA was carried out using a LifeECO thermocycler (Bioer, Hangzhou, China), which was programmed with specific cycling conditions. The quantification of the amplicons was subsequently performed using a Qubit fluorometer. The barcoded samples were aggregated to achieve an equimolar concentration and then purified using AMPure XP Beads. This process was then followed by a quality control check. A volume 50 fmol of the pooled sample was utilized for the preparation of library. The sequencing was carried out on a MinION MK1C (Oxford Nanopore Technologies, Oxford, United Kingdom) device using a flow cell FLO-MIN114, as indicated by the manufacturer’s protocol for a duration of approximately 48 h.

### 2.6 NGS data analysis

The minKNOW version 6.0.14 (Oxford Nanopore Technologies, Oxford, United Kingdom) was utilized to facilitate real-time analysis of base calling and data acquisition, with a minimum Q score of 9. The base calls generated were meticulously stored in FASTQ format. Subsequent to the completion of the run, an analysis was conducted on the EPI2ME software (Oxford Nanopore Technologies, Oxford, United Kingdom) (Miah et al., 2023). The taxonomic classification of the sequences was conducted using Kraken2 and Silva with utmost precision. The operational taxonomic unit (OUT) that resulted was then subjected to further downstream analysis via Microbiome Analyst 2.0 (McGill, Canada) (Dhariwal et al., 2017), where alpha diversity, beta diversity and linear discriminant analysis effect size (Lefse) plots were studied.

### 2.7 Swiss-roll preparation of intestinal tissue

Rodents were euthanized by cervical dislocation. The entire small and large intestines were excised and moistened with 1X phosphate-buffered saline (PBS). The ileum (from the small intestines) were isolated, cut into smaller segments, and aligned with the caudal end facing the researcher. To remove fecal matter, ice-cold 1X PBS was gently flushed through the lumen from the caudal end. After initial cleaning, tissues were immersed in fresh PBS, slit longitudinally from the caudal end, and flushed again with buffer. The Swiss-roll technique was used to visualize a large portion of the intestinal tissue in a single section (Moolenbeek and Ruitenberg, 1981). Cleaned segments were tightly rolled around a thin wooden stick with the mucosa facing outward, secured with stainless steel pins, and fixed overnight in Bouin’s solution. The next day, tissue dehydration began by placing samples in 70% ethanol at 4°C overnight. On the third day, samples were brought to room temperature, transferred to 90% ethanol for 1 h, then incubated twice in 100% ethanol for 2 h each. Clearing was performed with a 1:1 xylene–ethanol solution for 30 min, followed by two washes in xylene for 30 min each. Paraffin infiltration was done in three steps, 30 min each. Rolls were embedded in paraffin using a Thermo Scientific HistoStar workstation and stored at −8°C. Sections were cut at 5 μm using a Leica RM 2125 RTS microtome (Heidelberg, Germany) and mounted on 0.5% gelatin-coated slides.

### 2.8 Immunofluorescence

Slides were dewaxed in xylene (5 min, twice) and rehydrated through graded ethanol: absolute ethanol (3 min, twice), 95%, then 70% (3 min each), followed by PBS and distilled water. Antigen retrieval was done using citrate buffer. After three washes in PBS (5 min each), primary antibodies were applied and incubated overnight, at room temperature for NeuN (only brain slices), and at 4°C for CD8. The primary antibodies used were: anti-CD8 alpha (EPR21769, rabbit, Abcam, 1:2000), anti-Iba1 (rabbit, Wako, 1:2000), and anti-NeuN (mouse, EMD Millipore, 1:200). The next day, slides were washed in PBS and incubated with secondary antibodies: goat anti-rabbit Alexa Fluor^®^ 594 (1:300, Invitrogen A11012) and goat anti-mouse Alexa Fluor^®^ 594 (1:300, Invitrogen A11032). Sections were mounted using DAPI-containing medium (Abcam AB104139). All images were captured using an Olympus confocal microscope at 40 × magnification. Quantification of Iba1, CD8, and NeuN-positive cells was performed using ImageJ software as previously described (Nakhal et al., 2025a).

### 2.9 Statistical analyses

For the microbiome statistical analyses, we used the MDP module in the Microbiome Analyst 2.0 (McGill, Canada) (Chong et al., 2020). The data were filtered with a minimum count of 4 and a low variance filter of 10% and then normalized using Total Sum Scaling (TSS). Core microbiome analysis was examined using a heat map generated by 20% sample prevalence and 0.01% relative abundance. A comparison of alpha diversity measures (Cha01, Shannon, and Simpson) was conducted using a *post-hoc* pairwise comparison approach for multiple groups, with statistical analysis performed using Mann–Whitney/Kruskal-Wallis method. The Bray-Curtis indices were employed to study the beta diversity and it was then visualized by principal component analysis (PCA) plot (Abu et al., 2024). Permutation-Based Analysis of Variance (PERMANOVA) served as a statistical tool for conducting a comparative analysis of the beta diversity indices across a total of four distinct groups. The statistical significance of the observed results was determined by establishing a *p*-value cut-off point of 0.05 or less. The Linear Discriminant Analysis (LDA) was employed to assess the relevance or effect size of differentially abundant features. A threshold of 2 was established for the logarithmic LDA score of the discriminative features.

For the morphometric statistical analyses, the statistical program Sigma Stat 12 (SPSS Incorporated) was used. Comparisons between two groups were conducted using either the Student’s unpaired *t*-test or the Mann–Whitney test, depending on whether the normality test (Shapiro–Wilk) passed. For the Sholl dendritic intersection analyses, we performed a 2-way repeated measures ANOVA with treatment (dysbiosis vs. control) as the between-group factor and radial distance from the soma. Violation of the sphericity assumption for repeated measures was corrected using the Greenhouse–Geisser correction for degrees of freedom. At each distance interval between the control and AIGD groups, *t*-tests *post-hoc* with Bonferroni correction were performed.

## 3 Results

### 3.1 Validation of dysbiosis model

The presented gut dysbiosis model was rigorously validated through a multi-faceted approach. First, alpha diversity metrics—including microbial richness, evenness, and dominance—were assessed to evaluate intra-group diversity. Second, beta diversity analysis was performed to compare gut microbiota composition across experimental groups. Third, Linear Discriminant Analysis (LDA) scores were used to identify shifts in pathobionts and commensal microbiota. Finally, microbial dynamics were examined by quantifying the relative abundance of SCFA-producing families and species with known neuroprotective or neurotoxic properties For clarity, the following abbreviations are used throughout the manuscript to denote the experimental groups: Pre-C: control animal group before treatment; Post-C: control animal group after 14 days of the vehicle (normal saline) treatment; Pre-T: antibiotic-treated animal group before treatment; Post-T: 14 days of antibiotic-treated animals.

#### 3.1.1 Alpha diversity analysis

**Figure 2:**
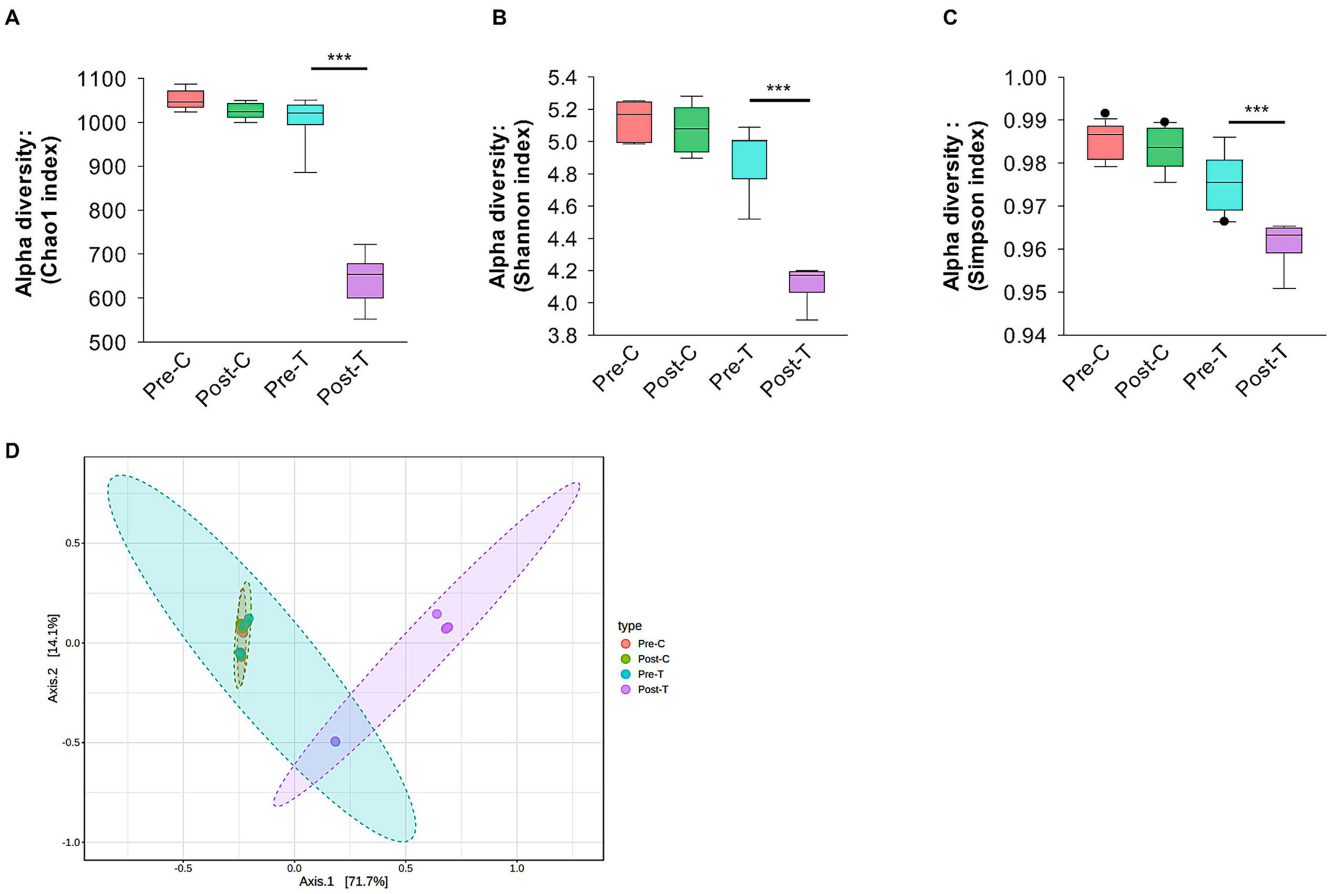
Alpha and beta diversity analysis. Alpha diversity analysis of gut microbiota using Chao1 **(A)**, Shannon **(B)** and Simpson **(C)** indices. **(D)** Beta diversity evaluation by Principal Corodinate Analysis (PCoA) based on Bray-Curtis distance matrix method. Alpha and beta diversity with *p* values less than 0.001 considered significant were calculated using Microbiome Analyst (Microbiome package 2.0).

Alpha diversity analysis was conducted to measure the richness, evenness, and dominance within a single microbial community. Specifically, the Chao1, Shannon, and Simpson indices were calculated. *Post-hoc* pairwise comparisons revealed that alpha diversity indices were significantly decreased in the Post-T group compared to the Pre-C, Post-C, and Pre-T groups (Chao1: Kruskal-Wallis, *p* < 0.001; Shannon: Kruskal-Wallis, *p* < 0.001; Simpson: Kruskal-Wallis, *p* < 0.001; Figures 2A–C). However, no statistically significant differences in microbial richness or evenness were observed between the Pre-C, Post-C, and Pre-T groups.

#### 3.1.2 Beta diversity analysis

Beta diversity metrics were used to assess compositional variations and dissimilarities in the gut microbiota across the experimental groups. Principal coordinates analysis (PCoA) and the Bray-Curtis distance method were applied at the species taxonomic level to visualize and quantify these differences. After 2 weeks of antibiotic treatment (Post-T), the gut microbiota composition became significantly distinct from both the Pre-T baseline group (PERMANOVA, *p* < 0.01, *r*^2^ = 0.56719, F statistic = 11.794; see Figure 2D) and both control groups (Pre-C, PERMANOVA, *p* < 0.01, *r*^2^ = 0.72776, F statistic = 26.732; Post-C, PERMANOVA, *p* < 0.01, *r*^2^ = 0.70659, F statistic = 24.082; see Figure 2D). In contrast, mice receiving normal saline (Post-C) showed no significant differences in microbiota composition compared to the Pre-C baseline (PERMANOVA, *p* = 0.922, *r*^2^ = 0.02716, F statistic = 0.27919; Figure 2D) or Pre-T (PERMANOVA, *p* = 0.649, *r*^2^ = 0.08556, F statistic = 0.84209; Figure 2D). These results suggest that the route of administration (oral gavage with saline) did not contribute to the distinct microbiota composition observed in the Post-T group. Prior to finalizing the experimental regimen, we tested three different concentrations of the antibiotic cocktail to identify a dose that reliably induced gut dysbiosis without compromising animal welfare. At the lowest dose (0.8 mg/mL vancomycin, 0.415 mg/mL clindamycin, 2.4 mg/mL meropenem), no significant alterations in alpha or beta diversity were observed, and dendritic morphology appeared unchanged. The intermediate dose (1.6 mg/mL vancomycin, 0.83 mg/mL clindamycin, 4.8 mg/mL meropenem) produced consistent and reproducible dysbiosis and was selected for the main experiments. In contrast, the highest dose (3.2 mg/mL vancomycin, 1.66 mg/mL clindamycin, 9.6 mg/mL meropenem) resulted in 50% mortality and was excluded from further use. These findings suggest a threshold effect in microbiota–brain interactions rather than a linear dose–response relationship (see Supplementary Figure 1).

#### 3.1.3 LDA scores of bacterial families: pathobionts vs. healthy flora

**Figure 3:**
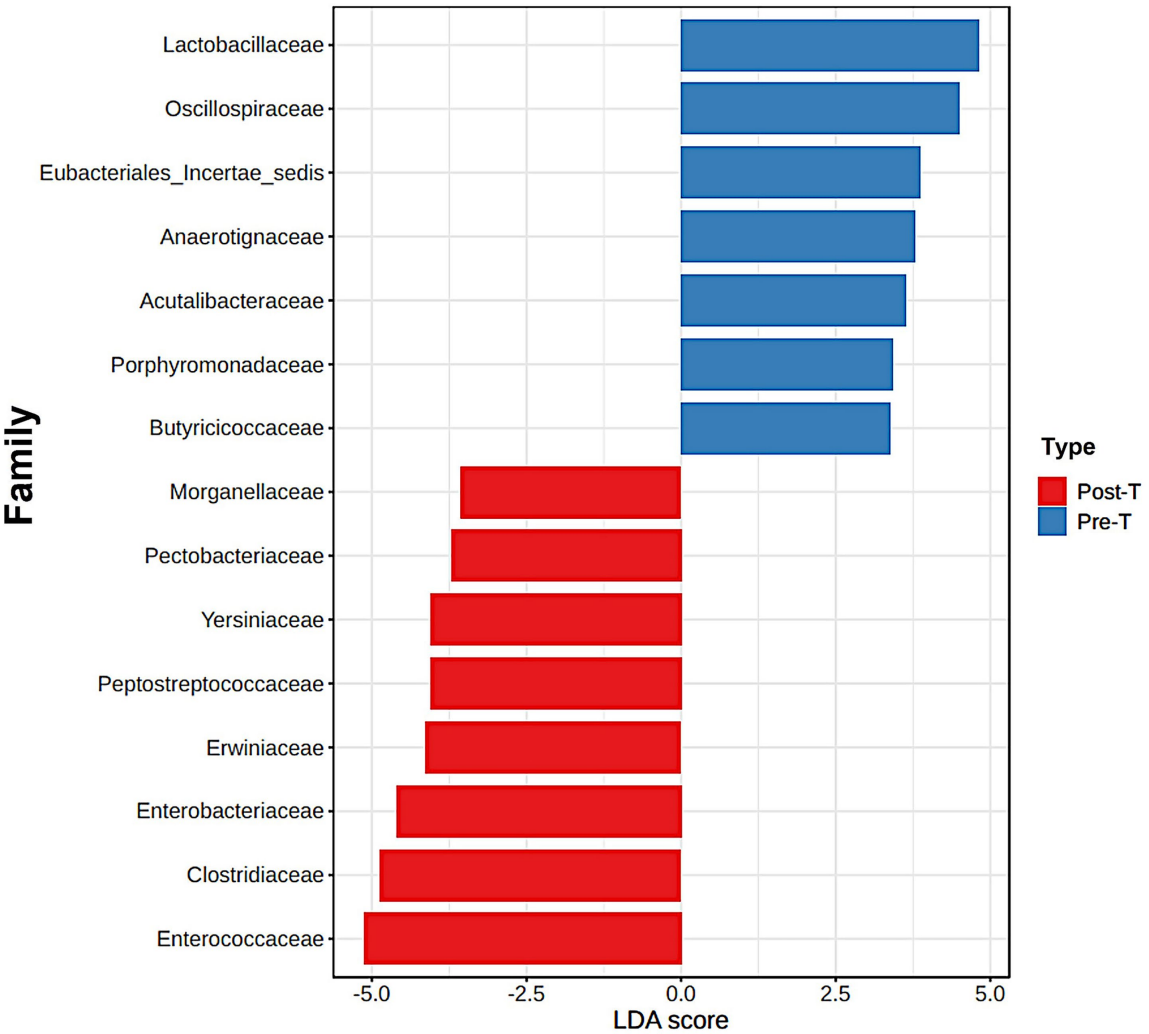
The differences between the Pre-T and Post-T groups were evaluated using the linear discrimination analysis effect size (LEfSe). The LEfSe method, utilizes a Kruskal-Wallis sum test and linear discrimination analysis (LDA) to identify significant differences in abundance and to estimate the effect size, respectively. The bar graph represents the LDA scores of 15 significant bacterial families. The colors indicate which group was more abundant than the other at family level.

The Linear Discriminant Analysis Effect Size (LEfSe) method was used to identify bacterial families that exhibited significant changes between the Pre-T and Post-T groups (Figure 3). LEfSe analysis revealed distinct differences in the abundance profiles of microbial families. In the Pre-T group, families associated with the healthy gut microbiota, such as *Lactobacillaceae*, *Oscillospiraceae*, *Butyricicoccaceae*, and *Porphyromonadaceae*, had LDA scores above 3.5. In contrast, several pathobiont-associated families, including *Enterobacteriaceae*, *Clostridiaceae*, *Enterococcaceae*, and *Erwiniaceae*, exhibited significant alterations between the Pre-T and Post-T groups, with LDA scores of −3.5, indicating a higher abundance in the Post-T group compared to the baseline Pre-T group.

#### 3.1.4 Relative abundance of the SCFA butyrate-producing families

**Figure 4:**
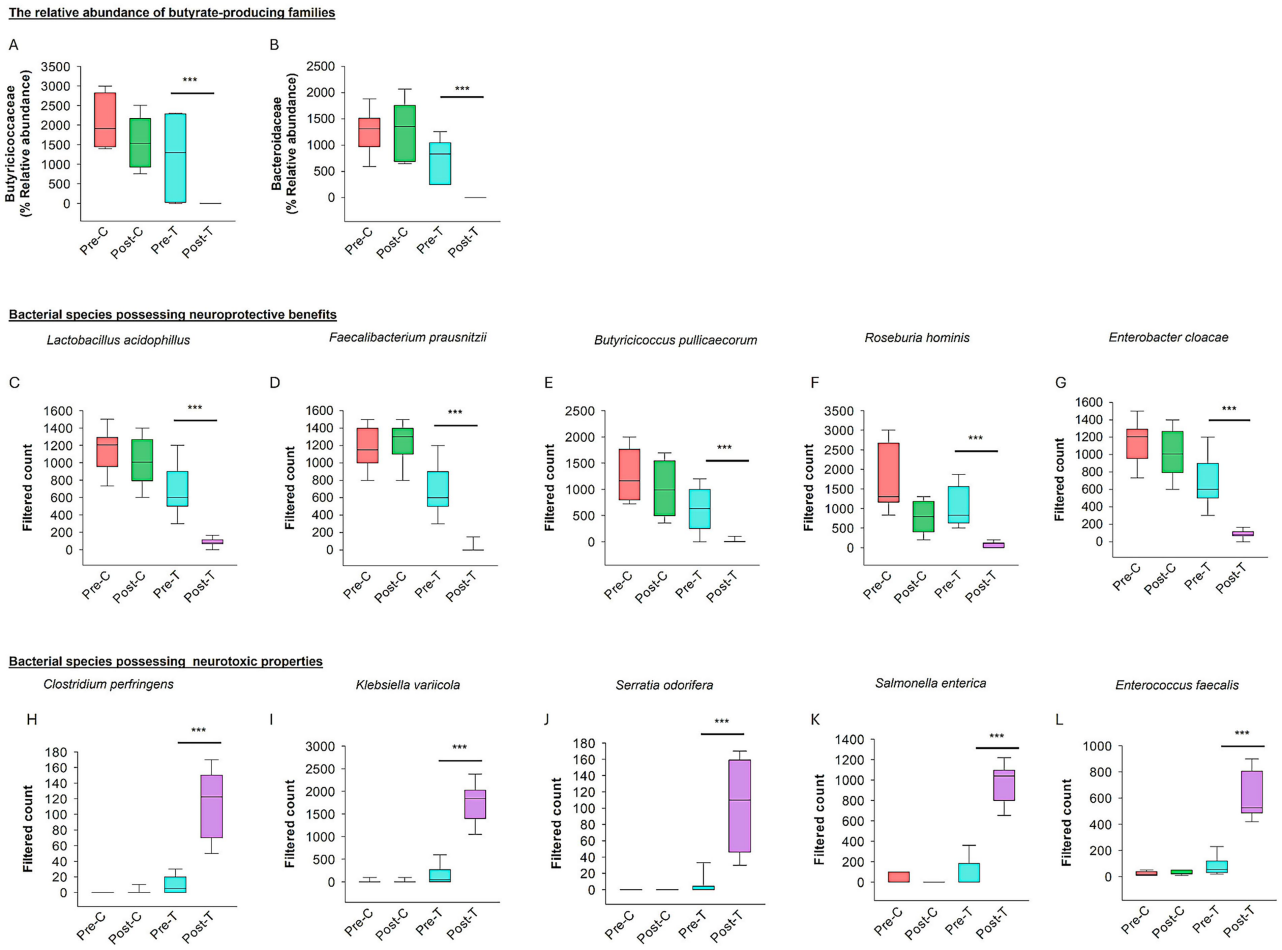
Effect of dysbiosis on neuroprotective and neurotoxic bacterial species. The relative abundance of the families producing butyrate (SCFAs) such as *Butyricicoccaceae* **(A)** and *Bacteroidaceae* **(B)**. **(C–G)** The box plots show the filtered count of bacterial species with neuroprotective properties among the four groups. **(H–L)** The box plots represent the filtered count of bacterial species that exhibit neurotoxic properties among the four groups. The data sets were obtained from Microbiome Analyst (Microbiome package 2.0).

The relative abundance of butyrate-producing families, including *Butyricicoccaceae* and *Bacteroidaceae*, was analyzed across the four experimental groups (Pre-C, Post-C, Pre-T, and Post-T). The Post-T group exhibited a statistically significant depletion of *Butyricicoccaceae* and *Bacteroidaceae* compared to all other groups (PERMANOVA test: *p* < 0.001; Figures 4A,B).

#### 3.1.5 Comparison of bacterial species associated with neuroprotective and neurotoxic properties

The relative abundance of bacterial species associated with neuroprotective and neurotoxic effects was assessed across the Pre-C, Post-C, Pre-T, and Post-T groups. A significant depletion in the relative abundance of neuroprotective species, including *Lactobacillus acidophilus*, *Faecalibacterium prausnitzii*, *Butyricicoccus pullicaecorum*, *Roseburia hominis*, and *Enterobacter cloacae*, was observed in the Post-T group compared to the Pre-C, Post-C, and Pre-T groups (PERMANOVA, *p* < 0.001; see Figures 4C–G). In contrast, the Post-T group exhibited a substantial increase in the relative abundance of neurotoxic species, including *Clostridium perfringens*, *Klebsiella* var*iicola*, *Serratia odorifera*, *Salmonella enterica*, and *Enterococcus faecalis* (PERMANOVA, *p* < 0.001; see Figures 4H–L). These findings indicate a distinct microbial profile associated with AIGD.

### 3.2 AIGD reduced dendritic length and complexity of stellate cells

**Figure 5:**
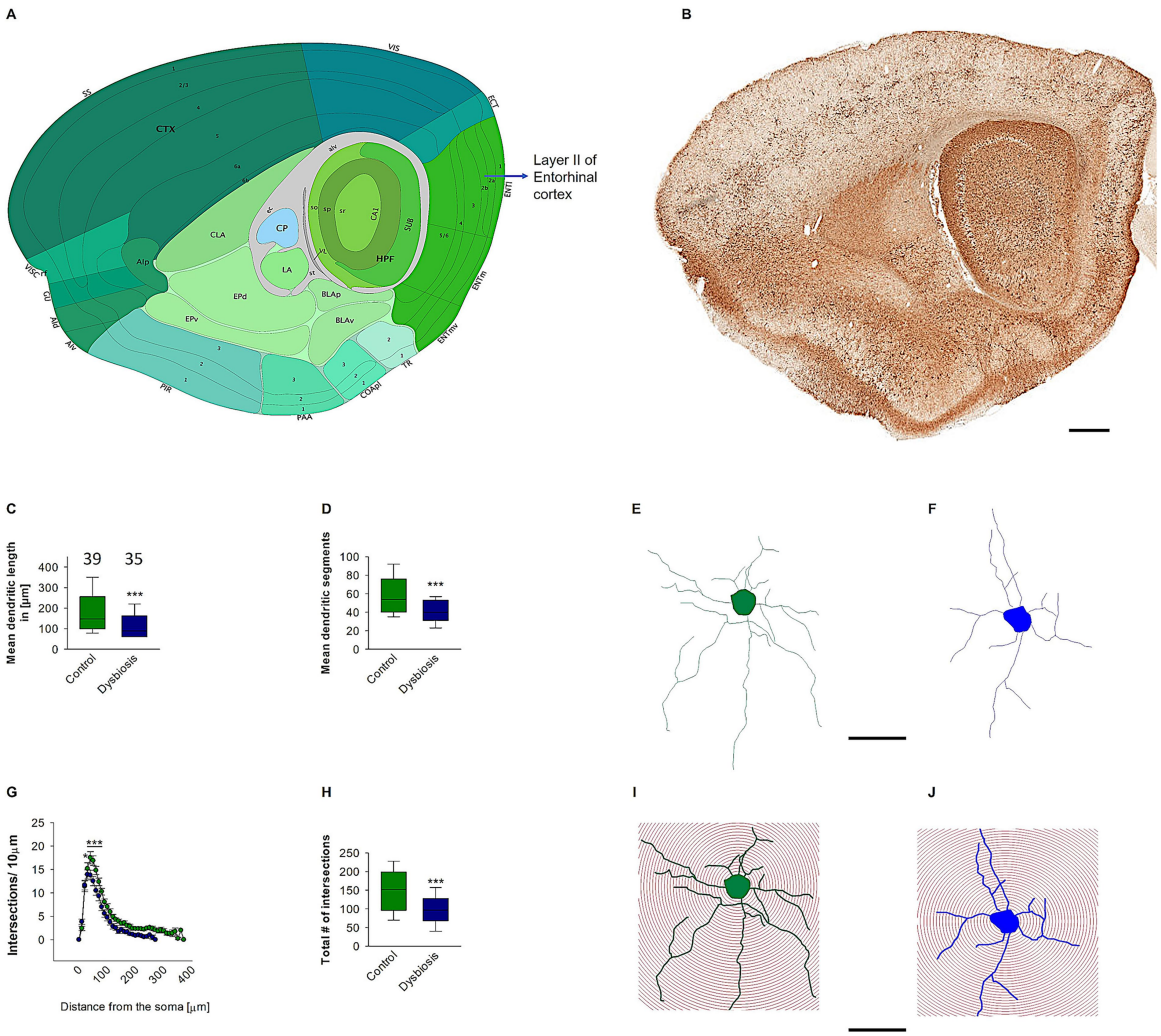
Effect of AIGD on stellate cells dendritic growth. **(A)** The adapted image from Allen brain mouse atlas illustrates the sagittal section of the mouse brain, with a marked emphasis on layer II of the medial entorhinal cortex, where stellate cells are localized. **(B)** The Golgi-stained sagittal section cut at the same depth as in **(A)** facilitates detailed visualization of the dendritic morphology. The scale bar in **(B)** is 500 μm. The boxplot in **(C)** represents the mean dendritic length of the stellate cells in the AIGD group and the control group. The boxplot in **(D)** shows the mean dendritic segments in the AIGD group compared to the normal control group. **(E,F)** are examples of images of a reconstructed stellate cell from the control group and the AIGD group, respectively. The graph in **(G)** involves a Sholl analysis, which compares the number of intersections between stellate cell dendrites in the AIGD and control groups at varying distances from the soma. The boxplot in **(H)** represents the mean total dendritic intersections between the control and AIGD groups. **(I)** An example of Sholl analysis conducted with an incremental radius of 10 μm from the soma of an stellate cell from the control group, and a similar example in **(J)** is taken from the AIGD group. The scale bar is 100 μm.

The present study hypothesized that the dendritic complexity and arborization of stellate and island pyramidal neurons in the mECII could be affected by AIGD. To test this hypothesis, morphometric analyses of reconstructed stellate neurons revealed a significant reduction in mean dendritic length in the AIGD group compared to the control group (Mann–Whitney test, *p* < 0.001; Figure 5C). A similar reduction in mean dendritic segments in the mECII of the AIGD group was also observed (Mann–Whitney test, *p* < 0.001; Figure 5D). However, the mean number of primary dendrites of stellate cells in the AIGD group did not differ from the control group (data not shown). To determine the specific dendritic compartment where the reduction in dendritic complexity occurred, a Sholl analysis was performed. This analysis revealed a significant decrease in the number of proximal dendritic intersections (between dendrites and concentric circles at 10-μm intervals) between 40 and 70 μm from the cell body in the AIGD group compared to the control group (Two-way repeated-measures ANOVA, *p* < 0.05, *p* < 0.001, *p* < 0.001, *p* < 0.001; Figure 5G). Additionally, the Sholl analyses showed a significant reduction in the total number of dendritic intersections in the proximal dendritic compartment of the AIGD group compared to the control group (Mann–Whitney test, *p* < 0.001; Figure 5H).

#### 3.2.1 AIGD does not affect island pyramidal neuronal morphology

**Figure 6:**
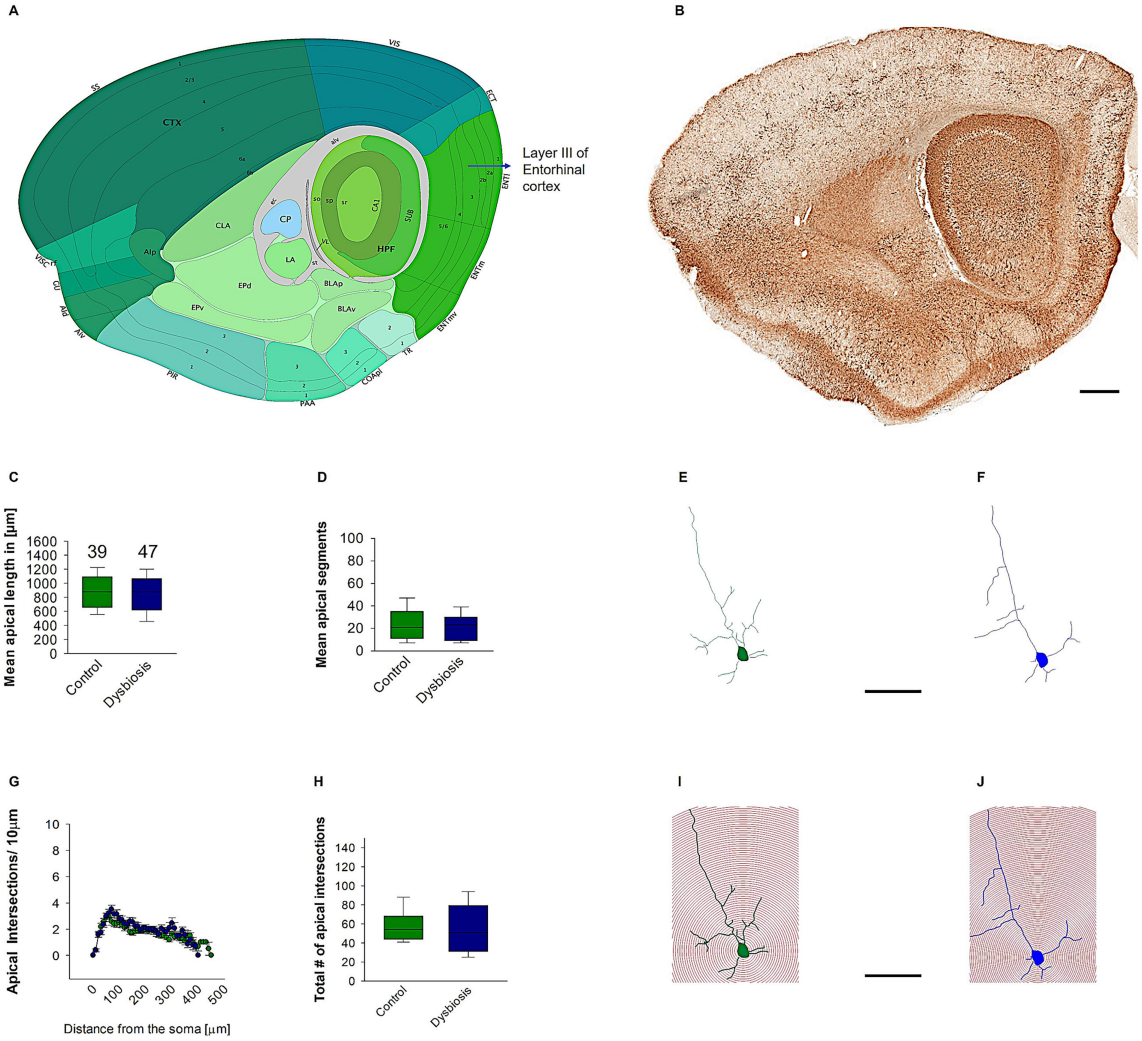
Effect of AIGD on apical dendrites of pyramidal island cells. **(A)** The adapted image from the Allen brain mouse atlas illustrates the sagittal section of the mouse brain, with a marked emphasis on layer II of the medial entorhinal cortex, where pyramidal island cells are localized. **(B)** The Golgi-stained sagittal section cut at the same depth as in **(A)** facilitates detailed visualization of the dendritic morphology. The scale bar in **(B)** is 500 μm. The mean dendritic length of the stellate cells in the AIGD group and the control group is presented in **(C)**. The mean dendritic segments in the AIGD group compared to the normal control group is presented in **(D)**. Examples of images of a reconstructed pyramidal island cells from the control group and the AIGD group are presented in **(E,F)**, respectively. The graph in **(G)** involves a Sholl analysis, which compares the number of intersections between stellate cell dendrites in the AIGD and control groups at varying distances from the soma. The boxplot in **(H)** represents the mean total dendritic intersections between the control and AIGD groups. **(I)** an example of Sholl analysis conducted with an incremental radius of 10 μm from the soma of pyramidal island cells in the control group is shown in **(J)**, and a similar example is shown in the AIGD group. The scale bar is 100 μm.

To determine the impact of AIGD on the morphology of pyramidal island cells, morphometric analyses were conducted. These analyses revealed that AIGD did not affect the mean apical dendritic length (Mann–Whitney test, *p* = 0.41; Figure 6C) or the mean apical dendritic segments (Mann–Whitney test, *p* = 0.61; Figure 6D) when compared to the control group. To further substantiate the finding that AIGD does not induce alterations in the dendritic complexity of apical dendrites in pyramidal island cells, a Sholl analysis was conducted. This analysis revealed no significant changes in the AIGD group compared to the control group (Two-way repeated-measures ANOVA, *p* = 0.58; Figure 6G). Moreover, the total number of dendritic intersections in the AIGD group did not differ significantly from the control group (Mann–Whitney test, *p* = 0.72; Figure 6H).

**Figure 7:**
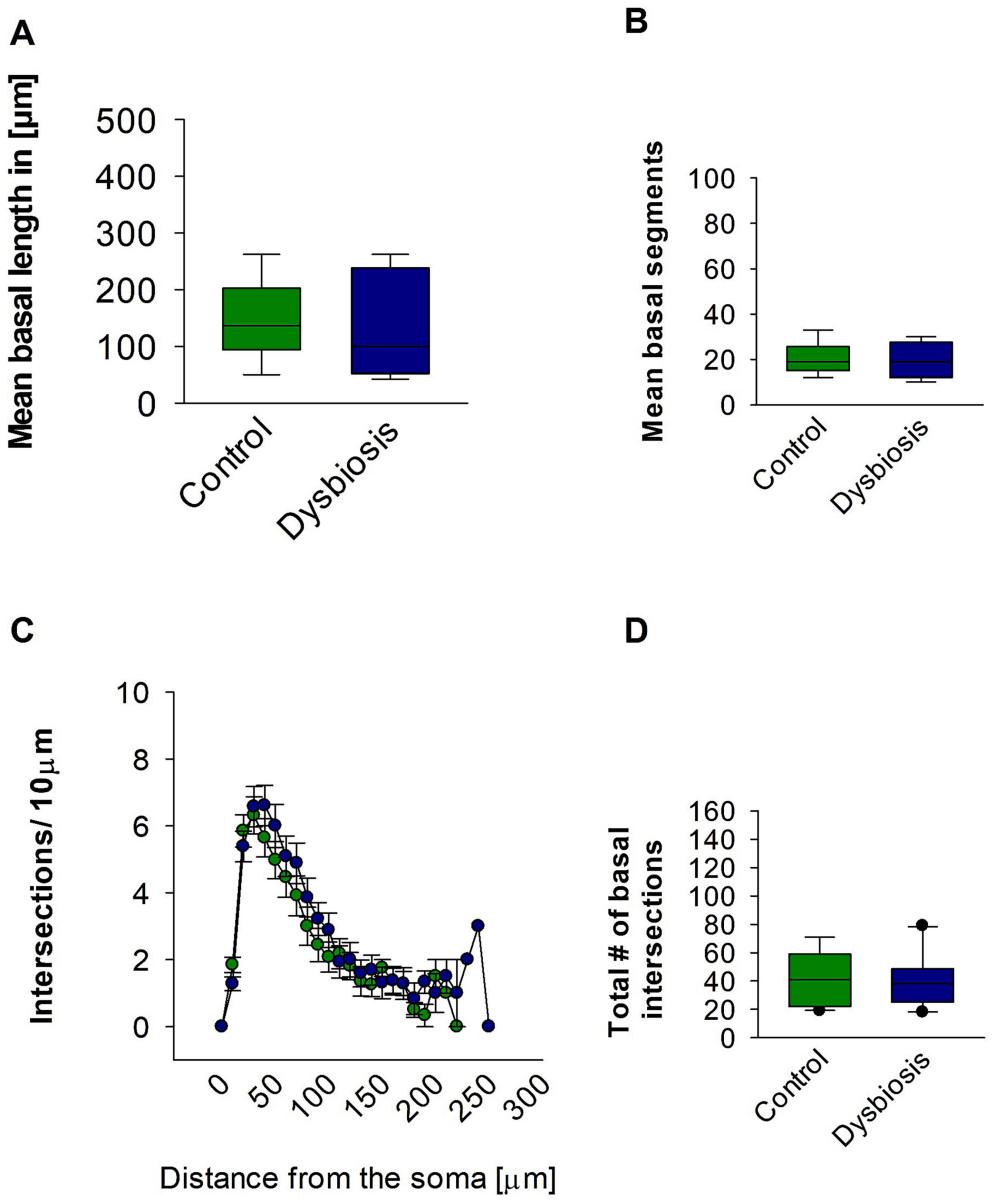
Effect of AIGD on basal dendrites of pyramidal island cells. **(A)** The boxplots shows the mean basal dendritic length and the mean basal dendritic segments is shown in **(B)**. **(C)** Sholl analysis of basal dendritic intersections of control and AIGD group. **(D)** The boxplot represents the mean total dendritic intersections between the control and AIGD groups.

Subsequent morphometric analyses of the basal dendrites of the pyramidal island cells also revealed no substantial alterations in their morphology. The mean basal dendritic length (Mann–Whitney test, *p* = 0.17; Figure 7A) and mean basal dendritic segments (Mann–Whitney test, *p* = 0.22; Figure 7B) remained unchanged in the AIGD group compared to the control group. Additionally, the Sholl analysis of the basal dendrites showed no significant change in the AIGD group compared to the control group (Two-way repeated-measures ANOVA, *p* = 0.75; Figure 7C). The total number of dendritic intersections in the AIGD group was also not significantly different from the control group (Mann–Whitney test, *p* = 0.41; Figure 7D).

### 3.3 Correlation between gut microbiota and dendritic morphology, and assessment of microglial activation

**Figure 8:**
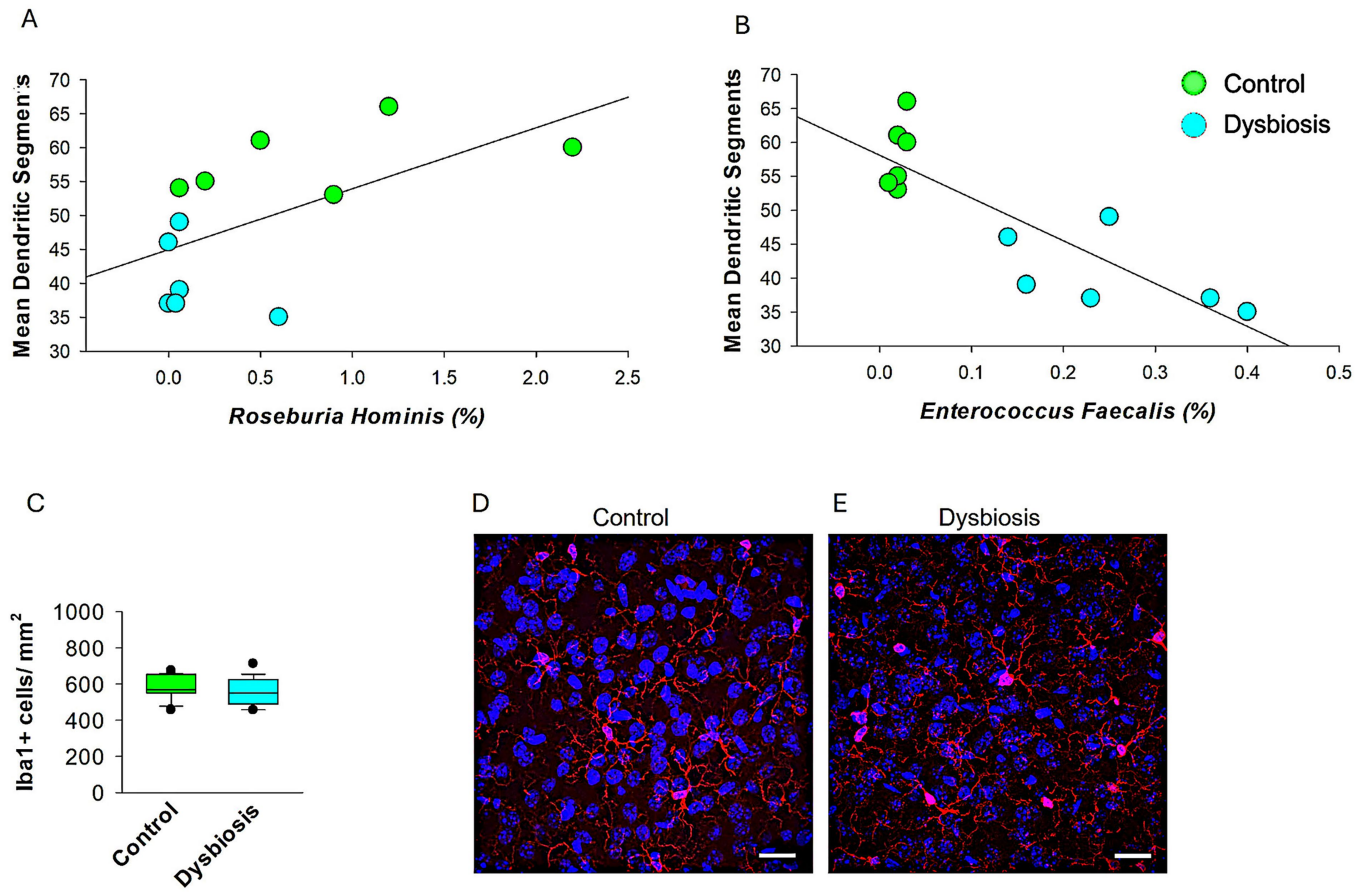
Microglial density in mECII of control and AIGD mice. **(A)** Spearman’s rank correlation analysis shows a significant positive association between *Roseburia hominis* relative abundance and dendritic segment number (*ρ* = 0.592, *p* = 0.0387), suggesting a possible neuroprotective relationship. **(B)** Spearman correlation analysis revealed a significant inverse relationship between *Enterococcus faecalis* abundance and dendritic segment number (*ρ* = −0.763, *p* = 0.00279, *n* = 12), indicating a possible link between dysbiosis and impaired neuronal morphology. Suggests a potential neurotoxic or dysbiosis effect of *Enterococcus faecalis* on dendritic structure. **(C)** Quantification of Iba1 + cell density (cells/mm^2^) in layer II of the MEC shows no significant difference between control and dysbiosis groups. Data represent mean ± SEM from 6 animals per group, with 24 sections analyzed per group. **(D,E)** Representative confocal images at 63x magnification of Iba1-immunolabeled microglia in the mECII of a control mouse **(D)** and a dysbiosis mouse **(E)**. Scale bar = 30 μm.

To explore potential mechanistic links between specific microbial taxa and neuronal morphology, we performed Spearman’s rank correlation analyses (Figure 8). We observed a significant positive correlation between the relative abundance of *Roseburia hominis* and the number of dendritic segments in stellate cells (Figure 8A, *ρ* = 0.592, *p* = 0.0387, *n* = 12), suggesting a possible neuroprotective role of this butyrate-producing species. In contrast, *Enterococcus faecalis* showed a strong negative correlation with dendritic segment number (Figure 8B, *ρ* = −0.763, *p* = 0.00279, *n* = 12), indicating a potential neurotoxic effect. These results link specific bacterial shifts associated with dysbiosis to morphological changes in mECII neurons.

To assess whether neuroinflammatory mechanisms contributed to the observed dendritic changes, we conducted IBA1 immunostaining to examine microglial activation in the mECII (Figure 8C-E). Microglia in both the control and AIGD groups exhibited similar morphology, with small somata and fine, and normal ramification processes. Moreover, no activated microglial phenotypes, such as highly ramified, amoeboid, or jellyfish-shaped morphologies (Vidal-Itriago et al., 2022), were observed in either the AIGD or control groups, further indicating the absence of overt microglial activation in the medial entorhinal cortex. Quantitative analysis of Iba1 + cell density revealed no significant differences between groups (Figures 8C, *p* > 0.05, Mann–Whitney test), suggesting that classical microglial activation was not present in response to AIGD. These results imply that the dendritic remodeling observed in stellate cells may occur through microglia-independent pathways, potentially mediated by microbial metabolites or circulating inflammatory signals.

### 3.4 Gut immune activation in response to dysbiosis

**Figure 9:**
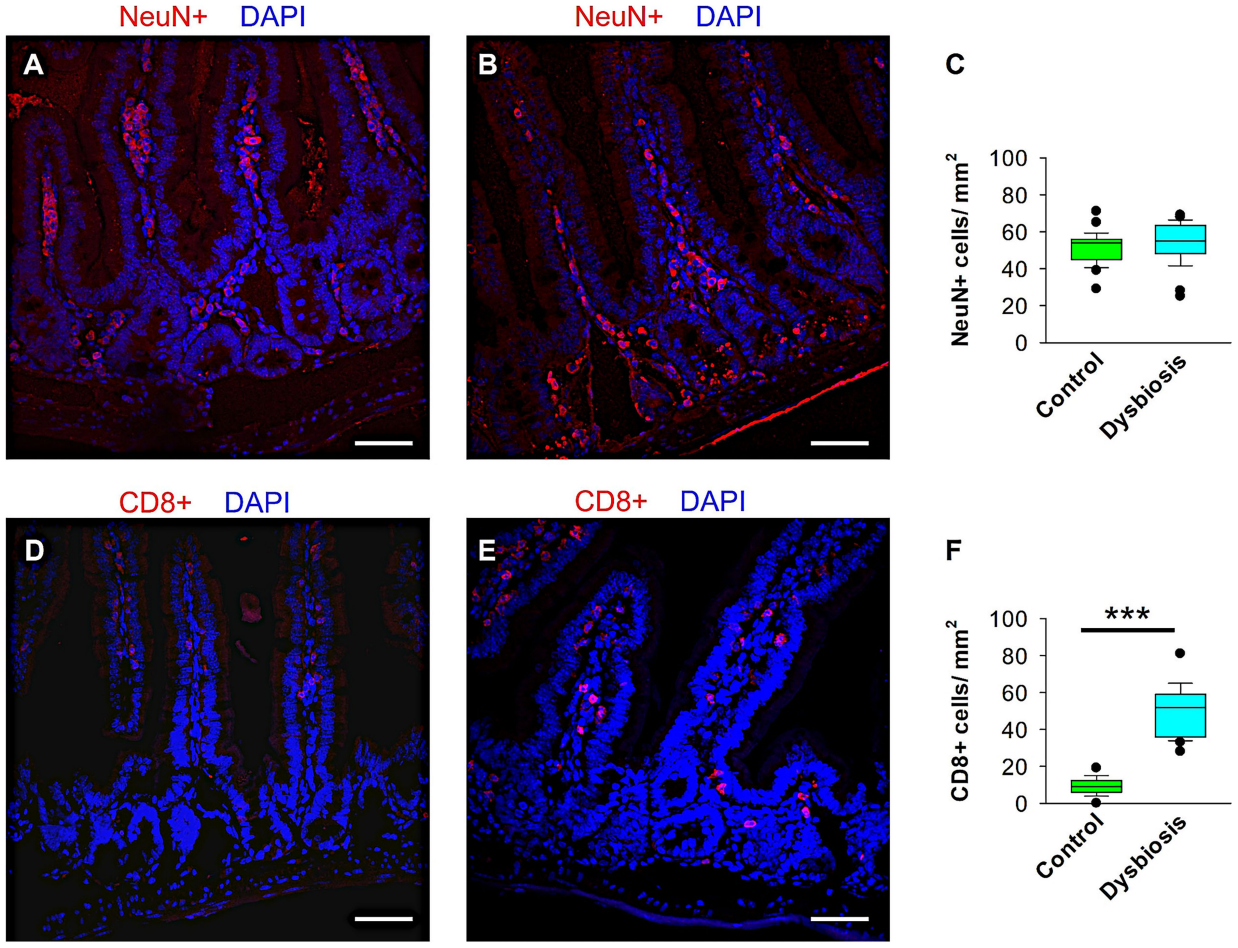
Quantification of CD8^+^ and NeuN^+^ cells in the small intestine of control and AIGD animal groups. **(A–B)** Representative confocal images at 40 × magnification showing NeuN^+^ cells (red) in the small intestinal of control **(A)** and AIGD **(B)** mice. **(C)** Quantification of NeuN^+^ cell density shows no statistically significant difference between groups. **(D–E)** Representative confocal images of CD8^+^ cells (red) in the small intestinal of control **(D)** and AIGD **(E)** mice. **(F)** Quantification reveals a significant increase in CD8^+^ cell density in AIGD mice compared to controls (*n* = 6 mice per group; ***p < 0.001). Nuclei are counterstained with DAPI (blue). Scale bar = 50 μm.

To assess whether antibiotic-induced gut dysbiosis (AIGD) altered intestinal neuronal architecture or triggered mucosal inflammation, we performed immunohistochemical analyses on small intestine tissue from control and AIGD-treated animals. Immunostaining with NeuN, a marker for mature neurons, revealed no significant difference in the number or organization of NeuN-positive enteric neurons between the two groups, suggesting that the overall architecture of the enteric nervous system (ENS) remained intact following antibiotic treatment (Figures 9A–C). In contrast, staining for CD8 revealed a marked increase in CD8-positive T lymphocytes in the small intestine in AIGD-treated animals compared to controls (Figures 9D–F). Quantitative analysis confirmed a statistically significant elevation in CD8 + cell density in the dysbiosis group (Mann–Whitney test, *p* < 0.001), indicating the presence of low-grade gut immune activation in response to dysbiosis. These findings support the hypothesis that microbial alterations can initiate peripheral immune responses even in the absence of overt ENS neuronal loss.

## 4 Discussion

The growing body of evidence underscores the critical influence of gut microbiota in neuronal development, synaptic plasticity, and overall brain function. Alterations in the microbial composition, particularly through AIGD, have been shown to profoundly impact neurological functions, potentially exacerbating or even contributing to neurodegenerative diseases. This connection has led to an increased focus on understanding the structure and function of mECII neurons, which are essential for higher cognitive processes, including spatial memory and navigation. Within mECII, stellate cells, in particular, have garnered attention due to their involvement in various neurological disorders, including Alzheimer’s disease (AD) (Vandrey et al., 2022). The present study investigates how AIGD impacts the dendritic morphology of mECII neurons, specifically the island and stellate cell populations. Our findings highlight a significant reduction in dendritic length, branching, and complexity of stellate cells, a population implicated in cognitive decline and neuropsychiatric disorders (Kordower et al., 2001). This selective vulnerability observed in stellate cells is consistent with findings in other models of neurodegeneration, such as the altered dendritic architecture of hippocampal pyramidal neurons in C58/J mice, an autism spectrum disorder (ASD) model (Barón-Mendoza et al., 2019). Interestingly, pyramidal island cells, in contrast, showed no significant morphological changes following AIGD, suggesting that different neuronal populations may be differentially susceptible to microbial disturbances. These results are in line with recent findings showing that antibiotic-induced dysbiosis alters the density and dendritic morphology of GABAergic interneurons in multiple brain regions, including the medial entorhinal cortex and hippocampus (Nakhal et al., 2025a), further supporting the role of the microbiota-gut-brain axis in shaping cortical microcircuitry. To further clarify the mechanistic link between gut dysbiosis and neuronal remodeling, we explored correlations between specific microbial taxa and dendritic architecture. A positive association between *Roseburia hominis* and dendritic segment number suggests a neuroprotective role for butyrate-producing bacteria, while a strong negative correlation with *Enterococcus faecalis* points to a possible neurotoxic influence. Interestingly, IBA1 immunostaining revealed no evidence of microglial activation in mECII, indicating that the dendritic alterations in stellate cells may occur independently of local microglial-mediated neuroinflammation and may instead be driven by microbial metabolites or circulating immune signals. Similarly, environmental factors such as early-life stress have been shown to reduce the number and size of GABAergic interneurons in limbic regions, including the amygdala and nucleus accumbens, highlighting the sensitivity of these neuronal populations to external perturbations (Aleksic et al., 2023; Nakhal et al., 2025b). The lack of change in pyramidal island cells may be attributed to distinct regulatory mechanisms governing dendritic plasticity within these cells, thereby reinforcing the concept of region-and cell-type-specific neuroplasticity.

The gut-brain axis and the role of microbiota-derived metabolites, such as SCFAs, in modulating cognitive function is an area of increasing interest. In addition to SCFAs, gut bacteria produce various neurotransmitters, including gamma-aminobutyric acid (GABA), serotonin, and dopamine, which can influence pyramidal cell excitability and synaptic plasticity (O’Mahony et al., 2015). These neurotransmitters modulate neural circuits by interacting with receptors on neurons, thereby impacting memory formation, learning processes, and overall brain function. Dysbiosis-induced alterations in microbial populations may disrupt this delicate neurochemical balance, further contributing to the observed morphological and functional changes in mECII neurons. Previous studies have shown that antibiotic treatment leads to reduced synaptic transmission in the hippocampal CA1 region (Çalışkan et al., 2022). Furthermore, oral antibiotic treatment in rodents has been associated with increased microglial density and altered synaptic activity, particularly at CA3-CA1 synapses, without affecting dendritic spine density (Basilico et al., 2019; Cordella et al., 2021). In line with these studies, our results indicate that AIGD leads the proliferation of proinflammatory bacterial families, including *Enterobacteriaceae*, *Clostridiaceae*, *Morganellaceae*, and *Pectobacteriaceae*, while simultaneously depleting SCFA-producing bacteria such as *Lactobacillaceae*, *Oscillospiraceae*, and *Butyricocaceae*. These shifts in microbial populations contribute to a dysregulated immune response, with proinflammatory gut bacteria increasing cytokine production, activating microglia, and promoting excessive synaptic pruning. Elevated proinflammatory cytokines like LIF have previously been shown to impair interneuron development, synaptic maturation, and dendritic architecture in the developing cortex (Engelhardt et al., 2018), reinforcing the detrimental impact of neuroinflammation on cortical microcircuit formation. Moreover, the depletion of SCFAs, key regulators of neuronal metabolism and plasticity, exacerbates dendritic growth deficits in stellate cells, resulting in destabilized synaptic architecture and impaired cognitive function. To further investigate whether antibiotic-induced dysbiosis triggered peripheral immune activation or altered enteric neuronal structure, we examined small intestinal tissues for signs of inflammation and ENS disruption. Interestingly, while NeuN immunostaining revealed no overt loss or disorganization of enteric neurons, consistent with a preserved gut neuronal architecture, CD8 immunostaining showed a marked increase in CD8-positive T cells in the lamina propria of dysbiotic animals, suggesting low-grade mucosal inflammation. This gut immune activation occurred despite the absence of local microglial activation in the brain, as shown by unchanged Iba1 staining in the mECII, supporting the idea that dendritic remodeling in stellate cells may be driven not by neuroinflammation per se, but by circulating immune factors or microbial metabolites originating from the gut. These findings underscore the importance of the gut-immune-brain axis and demonstrate that even in the absence of detectable enteric neurodegeneration or central microgliosis, dysbiosis can initiate peripheral immune signals that potentially influence brain development.

In addition to microbiome-induced inflammation, alterations in brain-derived neurotrophic factor (BDNF) signaling have been implicated in the pathophysiology of Alzheimer’s disease and other neurodegenerative conditions (Caffino et al., 2020). Antibiotic treatment has been shown to significantly reduce BDNF levels in animal models, impairing both spatial and long-term memory (Thabet et al., 2024). Reduced BDNF expression is linked to disrupted synaptic plasticity and dendritic complexity (Bistoletti et al., 2019; Suda and Matsuda, 2022). Our microbiome analysis reveals a marked decrease in the relative abundance of butyrate-producing bacteria, notably Butyricocaceae and Bacteriocaceae, which further supports the hypothesis that microbial dysbiosis negatively impacts BDNF signaling. This impairment in neurotrophic signaling contributes to the reduced number of dendritic nodes and intersections in stellate cells, which are critical for synaptic complexity and neuronal connectivity. The reduction in dendritic branching undermines the synaptic network, thereby limiting plasticity and long-term cognitive function. Emerging evidence from other brain regions shows that AIGD can reduce synaptic protein expression, such as synapsin-1 and PSD-95 (Çalışkan et al., 2022), and that microbiota restoration can reverse these deficits (Bairamian et al., 2022). Although we did not measure synapsin levels in mECII here, future studies assessing synaptic protein alterations in this region would help establish a direct link between dendritic remodeling and synaptic integrity following dysbiosis. In this context, the extracellular matrix protein Reelin—known for its role in neuronal positioning and synaptic plasticity—has also been implicated in structural remodeling during disease states, such as epilepsy, and may be influenced by neuroinflammatory processes triggered by gut dysbiosis (Leifeld et al., 2022). Further, certain gut microbiota species, such as *Faecalibacterium prausnitzii* and *Lactobacillus* spp., are known for their neuroprotective properties (Shandilya et al., 2022; Molska et al., 2024). *Faecalibacterium prausnitzii* has been shown to exert anti-inflammatory effects, enhance gut barrier integrity, and produce butyrate, which is essential for both gut and brain health (Fujimoto et al., 2013; He et al., 2021). The reduction of *Faecalibacterium prausnitzii* in response to dysbiosis compromises gut permeability, facilitating systemic inflammation and microbial translocation, both of which exacerbate neuroinflammatory and neurodegenerative processes. Our findings corroborate this concept, as the loss of *Faecalibacterium prausnitzii* following AIGD is linked to impaired cognitive function. Additionally, the depletion of *Lactobacillus acidophilus* and *Roseburia hominis*, both known for their neuroprotective roles, further supports the hypothesis that the gut-brain axis is disrupted by antibiotic-induced dysbiosis. These bacteria contribute to maintaining dendritic complexity and neuronal development by modulating BDNF signaling, which is essential for synaptic plasticity.

Conversely, the proliferation of neurotoxic bacteria, such as *Clostridium perfringens* and *Salmonella enterica*, following dysbiosis has been associated with neuroinflammation and the disruption of the blood–brain barrier. *Clostridium perfringens* secretes epsilon toxin (ETX), which impairs neuronal and microglial function (Linden et al., 2015; Wioland et al., 2015). Similarly, *Salmonella* LPS induces the expression of proinflammatory cytokines and neurotoxic effects on both pyramidal and GABAergic neurons in the hippocampus (Johansson et al., 2005). Our study observed a significant increase in the abundance of these toxigenic bacteria following antibiotic treatment, providing novel insights into their role in altering the dendritic structure of stellate cells. This neurotoxic influence may occur through the modulation of neurotransmitters, immune responses, and an imbalance of microbial metabolites, ultimately contributing to impaired neuronal development.

This study demonstrates the impact of AIGD on dendritic morphology in the mECII, but several limitations should be considered. First, while structural changes in stellate cells were observed, their functional consequences on synaptic activity and behavior remain unexamined. Electrophysiological studies and memory tests are needed to determine whether these morphological alterations translate into cognitive deficits. Second, although a gut-impermeant antibiotic cocktail was used, it is unclear whether similar effects would occur with other dysbiosis models, such as diet-induced microbiome alterations or germ-free conditions. Third, while microbiome analysis identified significant bacterial shifts, causal relationships between specific taxa and neuronal changes were not directly tested. Targeted microbial interventions, such as probiotics or metabolite supplementation, could clarify these mechanisms. Future research addressing these gaps will provide a more comprehensive understanding of the microbiota’s role in neuronal structure and function. Fourth, an additional limitation of our study is the potential systemic effect of meropenem, one of the antibiotics included in our dysbiosis model. While vancomycin and clindamycin are largely restricted to the gastrointestinal tract when administered orally, meropenem is partially absorbed into the bloodstream, which may influence systemic immune responses or circulating metabolites. Although our primary aim was to model gut-specific microbial disruption, we acknowledge that meropenem’s systemic absorption could contribute to off-target effects. Future studies employing fully non-absorbable antibiotics or alternative dysbiosis models are needed to disentangle gut-localized from systemic influences on brain structure and function.

## 5 Conclusion

This study highlights the impact of AIGD on the dendritic morphology of stellate cells in the mECII. Our findings demonstrate that AIGD selectively reduces dendritic length and complexity in stellate cells while sparing pyramidal island cells, suggesting differential neuronal susceptibility to microbiome disturbances. The observed depletion of neuroprotective bacterial species and expansion of proinflammatory taxa further supports a role for gut microbiota in modulating neuronal architecture. These results provide novel insights into the microbiota-gut-brain axis and its potential influence on spatial memory and cognitive function. Future studies integrating electrophysiological recordings, behavioral assessments, and targeted microbial interventions will be crucial for elucidating the functional consequences of these structural changes.

## Data Availability

The datasets presented in this study can be found in online repositories. The names of the repository/repositories and accession number(s) can be found at: https://data.mendeley.com/datasets/bh5mrcjdzf/1, 10.17632.
