# Supplementary material for: Selective vulnerability of stellate cells to gut dysbiosis: neuroanatomical changes in the medial entorhinal cortex
Source: Front Neuroanat. 2025 Aug 13;19:1589287. doi: 10.3389/fnana.2025.1589287 (PMC12380798; doi:10.3389/fnana.2025.1589287)
Supplement: Supplementary file 1 [file Data_Sheet_1.PDF]

Supplementary results to:

Selective Vulnerability of Stellate Cells to Gut Dysbiosis: Neuroanatomical Changes in the Medial Entorhinal Cortex

Ayishal B. Mydeen[1], Mohammed M. Nakhal[1], Faheema Nafees[1], Reem Almazrouei[1], Rasha Alkamali[1], Mahra Alsulaimi[1], Omar Aleissaee[1], Abdulrahman Alzaabi[1], Mohamed Alfahim[1], Hamad Almansoori[1], Shamsa BaniYas[1], Shaikha Al Houqani[1], Mariam Elkashlan[1], Safa Shehab[1], Mohammad I.K. Hamad[1*]

[1]Department of Anatomy, College of Medicine and Health Sciences, United Arab Emirates University, Al Ain, United Arab Emirates

Supplementary Fig. 1

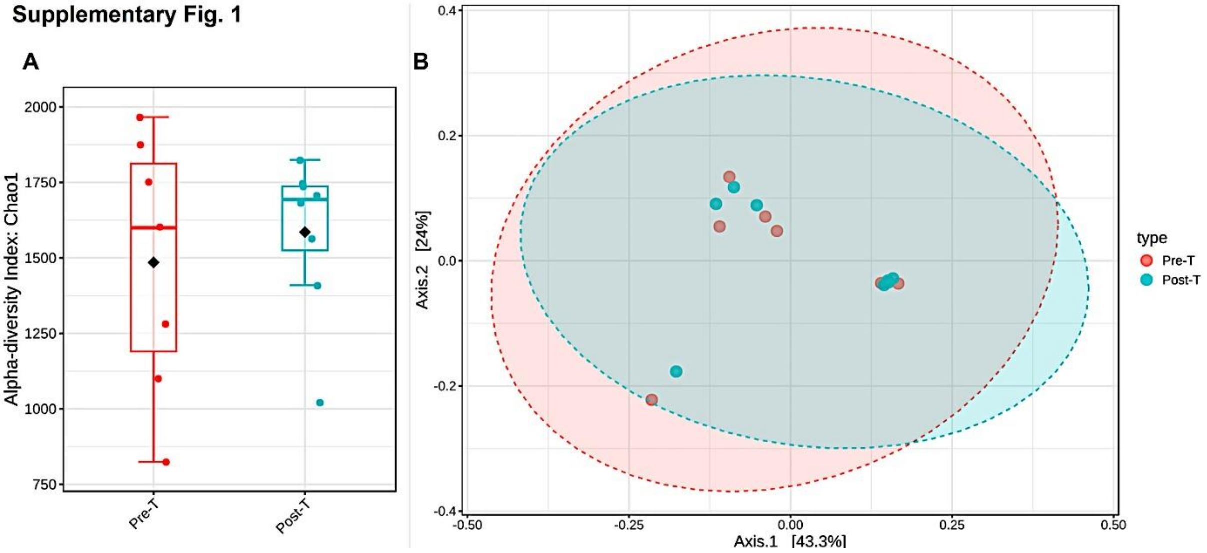

Supplementary Fig. S1. Alpha diversity analysis based on species richness revealed no significant difference in microbial diversity before and after treatment with a low-dose antibiotic regimen (0.8 mg/mL vancomycin, 0.415 mg/mL clindamycin, 2.4 mg/mL meropenem). **B.** Beta diversity analysis using Principal Coordinates Analysis (PCoA) based on Bray-Curtis dissimilarity showed overlapping microbial communities pre- and post-treatment. These results indicate that the administered low-dose antibiotic cocktail did not cause a substantial shift in gut microbial composition.
